# Computational methods for the identification of suicidal ideation: a systematic review

**DOI:** 10.3389/frai.2026.1704818

**Published:** 2026-01-22

**Authors:** Brahian Stiven Gil Arias, Juan Carlos Blandón Andrade, Grigori Sidorov, Alejandro Morales-Ríos

**Affiliations:** 1 Programa de Ingeniería de Sistemas y Telecomunicaciones, Universidad Católica de Pereira, Pereira, Colombia; 2 Centro de Investigación en Computación, Instituto Politécnico Nacional, Ciudad de México, Mexico; 3 Programa de Maestría en Ingeniería de Sistemas y Computación, Universidad Tecnológica de Pereira, Pereira, Colombia

**Keywords:** computational methods, artificial intelligence, natural language processing, suicidal ideation, systematic literature review

## Abstract

**Introduction:**

Suicide is one of the leading causes of death among young people, to the extent that in many countries it is considered a public health issue. It is important to attempt to reduce the growth of this trend, especially among susceptible individuals, considering that it increased because of the COVID-19 pandemic. Natural language processing (NLP) provides various tools that allow for the analysis of texts to predict the presence of suicidal ideation. This work aims to conduct a systematic literature review to extract the computational techniques for identifying suicidal ideation in texts written in natural language.

**Methods:**

The PRISMA 2020 method was used, which was divided into nine phases, and three inclusion criteria and two exclusion criteria were established for the selection of studies. The searches were conducted through high-level academic databases such as Scopus, IEEE Xplore, ACM Digital Library, Springer, and Web of Science. The risk of bias was assessed using AMSTAR 2. Potential biases identified include a lack of linguistic and cultural diversity and the predominance of data from social networks. A narrative synthesis was used to analyze and compare the findings qualitatively.

**Results:**

In the end, 25 studies related to computational methods for detecting suicidal ideation in texts written in natural language were identified. The techniques mainly focus on transformer-based models such as BERT and hybrid methods, which combine this architecture with neural networks such as CNN and LSTM. There are also approaches with hierarchical attention mechanisms. Some studies employed additional techniques such as feature extraction with TF-IDF and pre-trained embeddings to improve model performance.

**Discussion:**

Limitations in the evidence include the lack of linguistic and cultural diversity and the predominance of data from social networks. These results indicate that computational techniques have high potential to support early prevention strategies for suicidal ideation. However, expanding the diversity of linguistic contexts and improving understanding of the models among non-experts, such as physicians and other interested individuals, is necessary.

## 1 Introduction

Suicide is a relevant problem worldwide because it causes many deaths each year. In 2021, more than 48 thousand people died by suicide around the world, a figure that increased in 2022 to 49 thousand, which shows an increase of 2.6% in these 2 years (Centers for Disease Control and Prevention, 2023). A particularly vulnerable group is young people between 13 and 30 years old (World Health Organization, n.d.), who, due to a series of significant changes at this stage of life, are at greater risk of mental health problems, including the risk of suicide (Castellvi Obiols and Piqueras, 2018). Young people are also affected by socioeconomic factors and family interactions (Cañón Buitrago et al., 2018). It is important to emphasize that men are more prone to suicide, with a rate approximately four times higher than that of women (Centers for Disease Control and Prevention, 2023). According to the authors, the problem is complex and worrying; it can affect people of all ages and backgrounds, because the inconveniences of life can lead them to consider suicide as the only way out of their problems.

Many people who commit suicide act impulsively, while others go through a process of emotional decline that could be addressed with early prevention strategies (Tabares et al., 2020). Such interventions can dissuade people from carrying out these thoughts by providing the necessary help and support in a timely manner, considering the seriousness of an act such as suicide (Castellvi Obiols and Piqueras, 2018).

Technological advancement has facilitated the development of tools to process and analyze large volumes of texts, which has allowed the implementation of innovative techniques to identify warning signs in texts written in natural language. This favors intervention before self-destructive actions materialize (Andrade, 2022). These natural language processing (NLP) techniques can detect suicidal ideation from structured sources such as databases, semi-structured sources such as web pages, and unstructured sources such as free-form texts (social networks, medical records, online forums and blogs, text messages, emails, digital personal diaries) (Longhurst et al., 2014). This technological approach can be integrated as a fundamental part of broader suicide prevention and mental health promotion strategies. Furthermore, NLP-based computational methods are financially viable, since their implementation can be less expensive than other forms of early intervention (Blanca Casas and Guibert Reyes, 1998) and can significantly reduce response times.

The application of technologies such as natural language processing to detect suicidal ideation in natural language texts offers both challenges and opportunities for early suicide prevention. Cook et al. (2016) mention that NLP-based models achieved high predictive values using text messages or social media responses in tests applied to young people and adults. These models show great potential in quickly identifying people at risk of suicide, which can be a tool to identify suicidal ideation in non-clinical settings. Bejan et al. (2022) propose the use of NLP to improve the identification of suicidal ideation, demonstrating high accuracy in detecting this behavior. In addition, the studies present measures such as AUROC with a value of 98.6% to evaluate the effectiveness in identifying suicidal ideation, and a value of 97.3% was obtained in the identification of suicide attempts. Cao et al. (2022) emphasize the persistence of suicidal ideas and the relevance of social networks as platforms for interaction and personal expression in society, demonstrating the need to continue research and development of advanced NLP techniques. The authors agree that the extraction of suicidal ideation from written texts is a relevant field of research that requires further exploration. This systematic review examines computational techniques for identifying suicidal ideation in natural language texts, evaluates their performance across diverse contexts, and identifies opportunities for advancing suicide prevention technologies. Therefore, the following research question is posed: What computational techniques are used in the scientific literature to extract suicidal ideation from natural language texts?

The article’s structure is as follows: Section 2 presents the method used to conduct the review. Section 3 presents the results after applying the inclusion and exclusion criteria. Section 4 presents the discussion of the main findings, and Section 5 presents the conclusions.

## 2 Method

The method used for the systematic review is established by PRISMA (Preferred Reporting Items for Systematic Reviews and Meta-Analyses) (Page et al., 2021). The academic community accepts this method because it guarantees a good structuring of the information and contributes to the quality of the study. The method has several phases, which are described in this section.

### 2.1 Eligibility criteria

Inclusion and exclusion criteria are used to obtain relevant, high-quality research on computational techniques and approaches applied to analysis and detection of suicidal ideation. The inclusion criteria were defined as: (i) the content of the work must be related to computational techniques and approaches for the analysis and detection of suicidal ideation; (ii) the publication language must be English or Spanish; and (iii) the selected articles must be in indexed academic journals to ensure their quality and relevance. The exclusion criteria are: (i) incomplete articles will be discarded; (ii) articles published before January 1, 2018, will not be considered. Incomplete articles were discarded because they do not provide sufficient information to evaluate quality and reproducibility. The time limit ensures capturing only NLP methods consistent with recent advances in language modeling applied to mental health. This aligns with the method PRISMA 2020, which requires clear eligibility criteria (Page et al., 2021) and with the Handbook, which recognizes date restrictions and information availability as valid reasons to support scientific rationale (Higgins et al., 2024).

### 2.2 Information sources

Databases with high acceptance and reputation in the computer science academic community were considered. The selection considered the ranking of engineering journals in Google Scholar, which uses the H5 index (Scholar G, 2022) and the Scimago ranking (Rank and SJR, n.d.) which identifies high-quality databases based on their influence in the engineering field. Based on these rankings, the selected databases were Scopus, IEEE Xplore, ACM Digital Library, Springer, and Web of Science. In addition, we considered the recommendations given by Gamboa (2017).

### 2.3 Search strategy

A search equation was designed and used in each of the selected databases. Equation 1 includes terms related to suicidal ideation and computational topics:

$$\begin{array}{c} ("\text{suicidal}\;{\text{ideation detection}}^{"}\;{\text{OR}}^{"}{\text{suicidal thought}}^{"}\;{\text{OR}}^{"}{\text{suicidal intent}}^{"})\;\text{AND}\\ (\text{natural language texts OR natural language processing})\;\text{AND}\\ \left(\text{"computational methods"}\;{\text{OR}}^{"}{\text{Computational Techniques}}^{"}\;{\text{OR}}^{"}{\text{algorithm}}^{"}\right) \end{array}\;$$ (1)

Filters were applied to comply with inclusion and exclusion criteria: only articles published in English or Spanish after January 1, 2018, were selected to ensure recent information.

### 2.4 Selection process

A data extraction form was created to identify relevant variables in the collected studies. Two authors independently reviewed the studies. Disagreements were discussed, and a third author was consulted when necessary. Rejected studies were documented with reasons for exclusion to ensure transparency.

### 2.5 Data collection process

A careful and organized procedure was followed. First, a data collection form was used to record key variables and relevant information from each study, facilitating a systematic review. This process made understanding and analysis easier. Subsequently, data collection was performed for each study.

### 2.6 Data items

Key information was identified and recorded from each selected study, including: year of publication, authors’ names, type of texts analyzed, data source, sample size, NLP techniques and algorithms used, performance metrics, and results presented in each study to evaluate NLP model performance in detecting suicidal ideation.

### 2.7 Study risk of bias assessment

To assess the risk of bias in the included studies, two authors independently evaluated each study using predefined quality criteria. Any disagreements between reviewers were resolved through discussion until consensus was reached. The specific tool and classification results are reported in Section 3.3.

### 2.8 Synthesis methods

Study selection forms with defined variables and inclusion criteria were used. Results were organized uniformly for easy understanding and presented using graphs and tables, as recommended in the PRISMA 2020 method (Page et al., 2021). This captures essential metrics, such as accuracy and presentation of results. Additionally, a subgroup analysis was performed to analyze possible differences in computational methods. Overall, the method allowed for the synthesis of available evidence and the presentation of findings. Due to the methodological heterogeneity of the included studies regarding data sources, annotation schemes, operational definitions of suicidal ideation, types of computational models employed, and diversity of reported evaluation metrics, it was not possible to conduct a quantitative meta-analysis without compromising the validity of the results. According to the Cochrane Handbook (Higgins et al., 2024), meta-analysis should only be considered when studies are sufficiently homogeneous in terms of participants, interventions, and outcomes. Following PRISMA 2020 recommendations (Page et al., 2021) and the SWiM reporting guideline (Campbell et al., 2020), a narrative synthesis was conducted to compare patterns, identify methodological gaps, and highlight common trends, thus avoiding the statistical integration of non-homogeneous and non-directly comparable metrics.

### 2.9 Reporting bias assessment

The study selection showed potential biases, particularly in the data sources used and their origin. Indexed academic databases were critically analyzed, but this may lead to the exclusion of gray literature or other diverse sources. Furthermore, it was noted that most publications were in English and that the datasets came from digital platforms, such as Reddit and the social network X. This may limit the applicability of the results to other social and cultural contexts.

## 3 Results

### 3.1 Study selection

**Figure 1:**
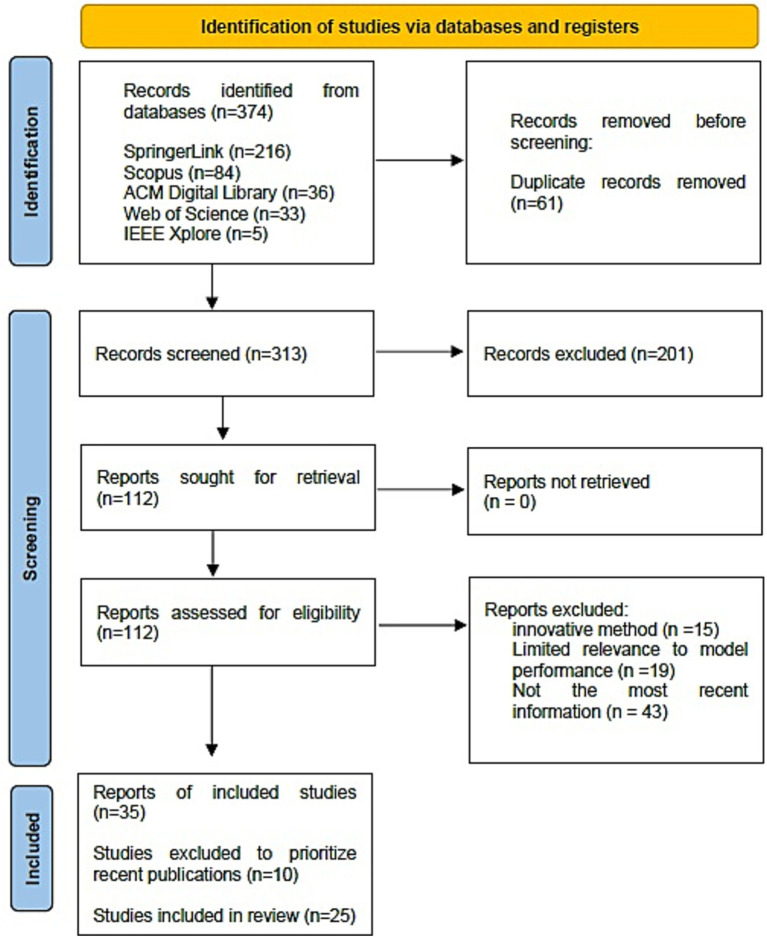
PRISMA-statement-based screening and filtering flow chart.

This phase was systematically conducted in multiple stages, ensuring compliance with predefined eligibility criteria. For a study to be eligible, it must address computational techniques and approaches for analyzing and detecting suicidal ideation, published in English or Spanish in an indexed academic journal. Initially, 374 papers were identified from five scientific databases (ACM Digital Library, IEEE Xplore, SpringerLink, Scopus, and Web of Science). After the duplication process, 61 duplicate records were eliminated. Two reviewers then worked independently to examine the titles and abstracts of the remaining 313 records, excluding 201 incomplete articles that did not meet academic publication standards. The same reviewers independently assessed the full text of the 112 articles that passed this initial review. In both selection stages, disagreements were resolved through discussion and, when necessary, by consulting a third reviewer. As a result of the systematic selection process, 35 articles were identified that met the inclusion criteria. Of these, 25 articles were selected for final analysis, discarding the 10 articles with the oldest publication dates, and prioritizing the most up-to-date information. The entire selection process was documented using the PRISMA 2020 flowchart (Page et al., 2021) as presented in Figure 1.

### 3.2 Study characteristics

This review included a total of 25 studies that analyzed the use of natural language processing (NLP) and machine learning techniques for the detection of suicidal ideation. The studies were geographically distributed: 11 were conducted in Asia, 7 in the Americas, 5 in Europe, and 2 in Africa. Regarding data sources, most studies used social media data (mainly Reddit and Social Network X), while others employed suicide notes, clinical content, voice data, or combined multiple data sources. The sample sizes in the analyzed studies presented a significant variability, with datasets ranging from approximately 500 samples in small datasets to more than 230,000 records in the most extensive studies. In terms of performance, the developed models showed wide variation in the evaluation metrics used, including precision, recall, F1-score, and accuracy. This variability reflects the differences between approaches, datasets, and objectives of each study.

**Figure 2:**
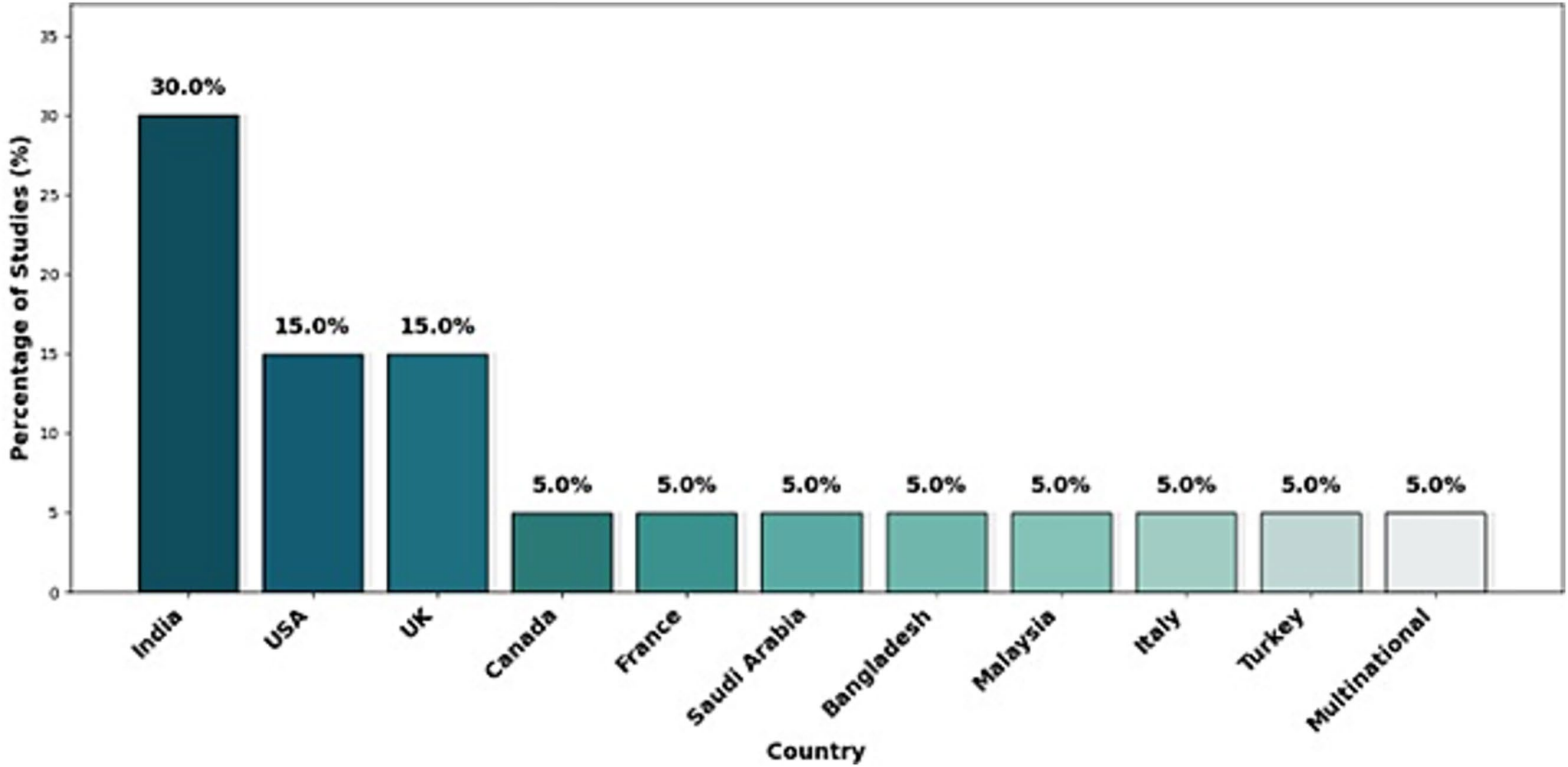
Geographical distribution of studies.

The geographical distribution of studies on detecting suicidal ideation using computational methods and natural language processing (NLP) shows that India leads with 30% of the studies, followed by the United States and the United Kingdom with 15% each. The remaining countries, including Canada, France, Saudi Arabia, Bangladesh, Malaysia, Italy, Turkey, and multinational collaborations, contribute 5% each. This analysis reflects the global diversity of research, with a significant focus on regions with high technological activity and interest in mental health, specifically in the search for a technological solution that can contribute to reducing suicide, as presented in Figure 2.

#### 3.2.1 Detected technologies

Long Short-Term Memory (LSTM) networks are extremely valuable tools for detecting suicidal ideation, due to their ability to model text sequences while preserving long-term dependencies. Due to this particularity, they have been widely used in research on texts linked to mental health problems. Schoene et al. (2023b) examined the effectiveness of hierarchical recurrent neural networks of different scales to classify suicide notes, highlighting the ability of LSTMs to identify important linguistic patterns in texts related to the concept of suicide. Their findings show the value of these architectures in natural language processing applications focused on the early detection of suicidal ideation.

The implementation of Bidirectional LSTM (Bi-LSTM) has improved the optimization of context representation in both directions of a text. Xie-Yi (2024) used a Bi-LSTM model with an attention mechanism to identify relevant keywords in posts on social network X, which facilitated the detection of risk factors related to suicidal ideation. The model achieved an F1-score of 95% using Word2Vec embeddings and showed that the attention mechanism helps to interpret the model’s decisions by highlighting meaningful words. In addition, it was demonstrated that Bi-LSTM models outperform conventional LSTM architectures by leveraging contextual information in both directions, providing better performance in text categorization tasks (Kumar et al., 2023; Li, 2025).

Convolutional neural networks (CNNs) have proven effective in identifying suicidal ideation on social media. Yao et al. (2020) developed a machine learning model to recognize signs of suicidal ideation in Reddit users with opioid use disorders. They found that CNNs achieved optimal performance with an F1 score of 96.6% in categorizing texts with suicide risk, highlighting the relevance of neural networks in automated language analysis in digital communities.

Anika et al. (2024), proposed a hybrid approach that combines a bidirectional gated recurrent unit (BiGRU) with CNN by analyzing multiple data sources and managed to capture complex patterns in social media texts. As a result, the model’s performance improved, and the classification accuracy of the analyzed texts increased.

Bidirectional Encoder Representations from Transformers (BERT)-based models have proven highly effective in detecting suicidal ideation. Boonyarat et al. (2024) optimized a Leveraging Enhanced BERT model for analyzing Thai text and developed a dataset consisting of 2,400 manually labeled tweets as part of the study. Using this methodology, they achieved an F1-score of 93% in predicting suicidal ideation and 88% in emotion classification. Furthermore, they identified a 40.97% increase in suicidal posts during the COVID-19 pandemic, highlighting the importance of employing automated tools for mental health monitoring on social media. On the other hand, Ezerceli and Dehkharghani (2024) implemented transformer-based deep neural networks to analyze suicidal ideation in different datasets. The model achieved an F1 score of 97% on the Suicide Detection set and 75% on the Corpus for the Evaluation and Analysis of Suicidal Expressions (CEASEv2.0). As part of their proposal, they propose an automated system that would help mental health professionals more quickly identify at-risk individuals, allowing them to act promptly and prevent potential crises.

Transformer-based models have been widely used to interpret depressive and suicidal behavior. Malhotra and Jindal (2024) present a transformer-based approach to analyze behavioral patterns in social media. They employ artificial intelligence techniques to facilitate model decisions in detecting suicidal behavior. Kodati and Tene (2024) show a model that combines dynamic context masking with convolutional neural networks to detect suicidal threats in social media and suicide notes. The model achieved high levels of accuracy in assessing the severity of suicidal ideation. Kumar et al. (2023) present a model based on RoBERTa and Bi-LSTM technologies to identify signs of depression in English and Arabic from social media, validating its effectiveness on multiple datasets.

Ensemble methods are effective in detecting suicidal ideation from social media because they combine multiple models to improve the accuracy and robustness of predictions. Bokhari et al. (2024) propose an approach to assess suicide risk, integrating classifiers such as Random Forest, Gradient Boosting, Support Vector Machine, k-Nearest Neighbors, and Naive Bayes, as well as a neural network together, thereby achieving an accuracy of 91% in risk prediction. Gorai and Shaw (2024) built an ensemble model that fuses BERT with convolutional neural networks (CNNs) to analyze social media posts. They leverage BERT’s ability to capture language context and CNN’s efficiency in detecting key text patterns. Mirtaheri et al. (2024) show a hybrid model that combines temporal convolutional networks (TCNs) with self-attention; this design optimizes the detection of suicidal ideation in social media. This allows for identifying deep semantic relationships and facilitates the analysis of large volumes of data. Renjith et al. (2022) implement an architecture that combines LSTM, CNN, and attention mechanisms, achieving more accurate classification of posts with suicidal ideation content by capturing contextual dependencies and assigning higher weights to key terms within the text.

Graph Neural Networks (GNNs) are used to analyze semantic relationships in texts related to suicidal ideation. Schoene et al. (2023a) uses GNN to classify posts with suicidal content on the social network X, thus identifying text patterns. Ahmed et al. (2023) uses Graph Attention Networks (GAT) to improve text classification and suicidal ideation detection by capturing meaningful connections between words and contexts.

Generative Adversarial Networks (GANs) are commonly used to balance datasets in suicidal ideation detection, improving data representation and reducing bias in classification models. Kancharapu and Ayyagari (2024) use GANs to generate high-quality synthetic data, which helps optimize the detection of suicidal posts on social media, thus improving the performance of machine learning models.

Large Language Models (LLMs) have proven helpful in predicting mental health conditions from online textual data. Xu et al. (2024) present Mental-LLM, an LLM-based model that, thanks to instruction-based fine-tuning, significantly improves the accuracy in predicting mental health problems compared to zero-shot and few-shot learning approaches. Ghanadian et al. (2024) use synthetic data for suicidal ideation detection, combining generative AI models with social factors extracted from psychological studies, thus seeking to improve the diversity and representativeness of the data.

In addition to Deep Learning architectures, some studies use traditional Machine Learning approaches for the detection of suicidal ideation. Vioulès et al. (2018) present a method to identify in real time posts with suicidal content on the social network X, they combine natural language processing techniques with traditional classification models, which allowed to detect abrupt changes in the linguistic behavior of users, thus offering a valuable tool for suicide prevention on social networks. Chatterjee et al. (2023) analyze posts on the social network X using machine learning models and demonstrate that including multiple types of information, such as text, images, and interaction data, significantly improves the early detection of risk signals.

Other authors prefer hybrid approaches that integrate multiple forms of information, such as text and speech analysis, combined with deep learning models that can be effective for the detection of suicidal ideation. Belouali et al. (2021) analyze the integration of acoustic and linguistic features to identify suicidal ideation in US veterans using a machine learning classifier that fuses information from speech and text. Priyamvada et al. (2023) propose a hybrid model to analyze social media posts through convolutional neural networks (CNNs) and LSTM networks. They use deep learning techniques and word embeddings with interesting results in detecting linguistic patterns associated with suicidal ideation. Qorich and El Ouazzani (2024) apply large-scale language models in this field. It is a hybrid model that combines CNN and BiLSTM with pre-trained embeddings such as Word2Vec, FastText, and GloVe, thus classifying suicidal posts on Reddit by integrating semantic and contextual analysis. Finally, Tlachac et al. (2022) present a rapid detection system for suicidal ideation in university students. They use the VGG model for voice analysis and BERT for text processing. It is a multimodal approach, which improves the accuracy in identifying at-risk users and generating active responses in mobile applications.

Audio-based models are an alternative for detecting suicidal ideation in contexts where data is limited. Pillai et al. (2024) studied the generalization capacity of these models by testing them on four datasets; however, they found difficulties in adapting them to different domains. To address this problem, they propose the sinusoidal similarity sub-sampling (S3) method, which obtained promising results in scenarios with variations in data distribution. The results highlight the importance of voice analysis and the need for adaptation techniques to improve the detection of risk patterns.

**Figure 3:**
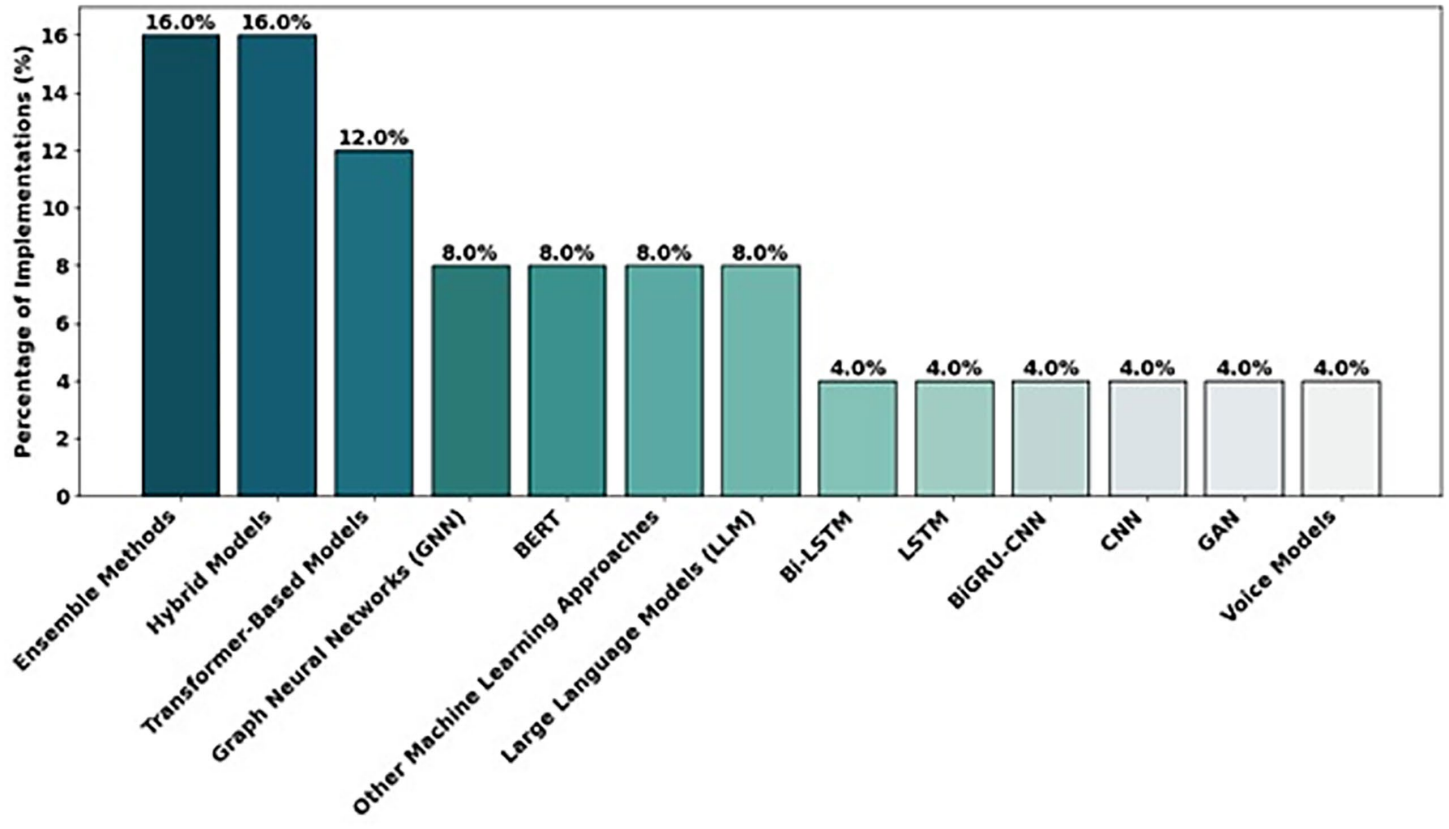
Distribution of studies according to their architectures.

The distribution of the different architectures used in the analyzed studies shows a greater use of ensemble methods and hybrid models (multimodality, NLP, and voice), compared to other approaches such as generative adversarial neural networks (GANs) or Bi-LSTM and CNN-based models. The distribution of the architectures is presented in Figure 3.

**Figure 4:**
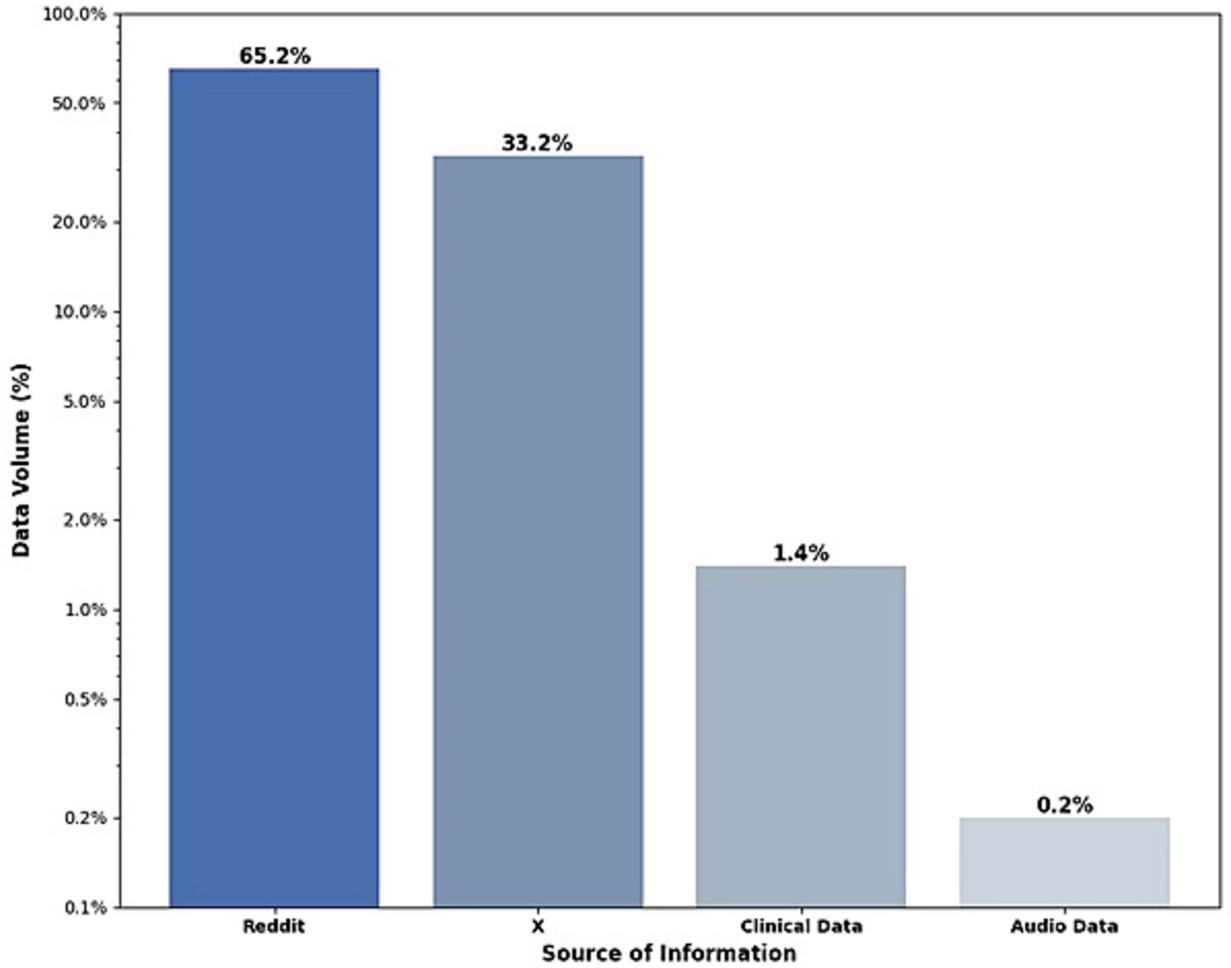
Distribution of data source used.

Figure 4 presents the distribution of data sources used in the analyzed studies. Reddit is the primary source, representing 65.2% of the total data volume, followed by X with 33.2%. In contrast, clinical and audio data have significantly lower representation, with 1.4 and 0.2%, respectively.

**Figure 5:**
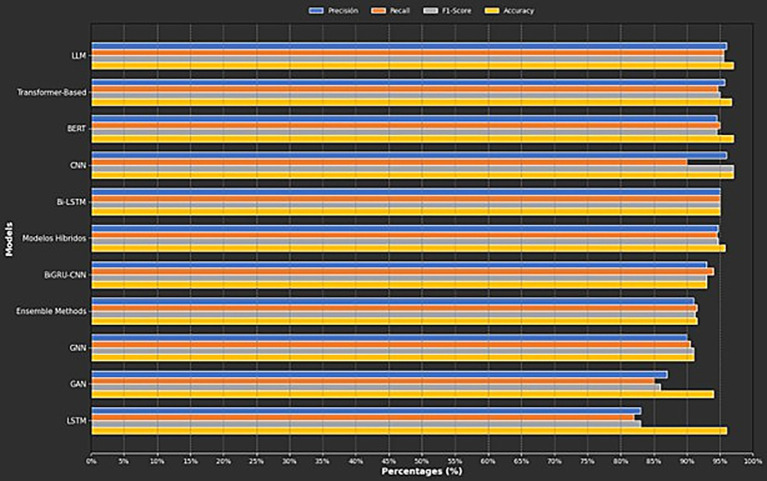
Metrics by category.

Four key metrics are used to analyze the performance of different computational approaches in detecting suicidal ideation: Accuracy, precision, recall, and F1-score. Accuracy indicates the percentage of correct predictions from the total number of analyzed cases. Precision reflects how many of the positive detections correspond to instances of suicidal ideation, helping to reduce false positives. Recall measures the model’s ability to identify all positive cases, minimizing false negatives. The F1-score assesses the balance between Precision and Recall, providing an overall average of model performance. Figure 5 presents the average values ​​of these metrics for each category of models analyzed in the literature.

### 3.3 Risk of bias in studies

The risk of bias in the included studies was assessed using the AMSTAR 2 tool (Shea et al., 2017), which allowed identifying methodological strengths and weaknesses in each work. The studies were classified as low, moderate, or high risk of bias. Among the main bias factors detected were the lack of blinding in the methods used, the selection of unbalanced data, and the absence of justification in the criteria used to filter or select the final data for the analysis. For example, some studies that worked with social media data presented language-related biases, since most of the publications were in English, which limits the applicability to other cultural contexts. Despite these limitations, cross-validation, techniques to avoid overfitting, and the participation of multiple reviewers during the development and evaluation of the models were used to ensure that the studies had a low risk of bias.

### 3.4 Results of individual studies

This section identifies how each study contributes to the overall analysis, facilitating comparisons between approaches, performance metrics, and technologies employed. Table 1 presents a summary of the study’s key findings.

**Table 1:** Synthesis of information.

Authors, year	Study population	Dataset size	Methods used	Metrics	Language processed
Ghanadian et al. (2024)	UMD real data, 9 GLLM synthetics, human-labeled test set, and augmented datasets.	100,000 real posts (UMD); 9 synthetic datasets (size not specified); combined datasets with up to 30% UMD.	Synthetic data generation with GLLMs (ChatGPT, Flan-T5, Llama) and fine-tuning of ALBERT/DistilBERT with real + synthetic data.	F1-score: 82% (synthetic only), 87% (30% UMD + synthetic); Accuracy: 82%	English
Schoene et al. (2023a)	Tweets related to suicide	112,969 tweets (3,977 annotated, 19,885 total labels; split: 80% train, 10% val, 10% test)	Feature GCN	Precision: 91%, Recall: 91%, F1 Score: 91%	English
Vioulès et al. (2018)	10 Twitter users with suicidal history, tracked for behavioral change.	11,000 tweets (1,000 pre-change + 100 post-change per user; 10 users)	Martingale Framework	—	English
Schoene et al. (2023b)	Study population: 211 genuine suicide notes (GSN) written by individuals who died by suicide.	Data: 633 (10-fold cross-validation); 5,004 (80% train, 10% val, 10% test).	Dilated LSTM with ranked units	Precision: 96%, Recall: 96%, F1-score: 96.1%	English
Renjith et al. (2022)	Reddit users (University of Maryland Reddit Suicidality Dataset)	69,600 posts (Training: 55,680 posts—80%; Testing: 13,920 posts—20%)	LSTM-Attention-CNN combined model	Precision: 91.6%, Recall: 93.7%, F1-Score: 92.6%, Accuracy: 90.3%,	English
Belouali et al. (2021)	US veterans meeting the Center for Disease Control’s criteria for Gulf War Illness	588 audio recordings (504 non-suicidal, 84 suicidal) from 124 veterans.	Random Forest Classifier (Analysis caustic y linguistic)	Accuracy: 72%, Precision (PPV): 32%, Recall Sensitivity: 84%, F1-Score: 45%, Specificity: 70%, AUC: 80%.	English
Priyamvada et al. (2023)	Twitter users who post suicidal and non-suicidal tweets.	10,000 tweets, with 5,126 labeled as suicidal ideation and 4,833 labeled as non-suicidal	Stacked CNN—2 Layer LSTM	Accuracy: 93.92%, Precision: 93.43%, Recall: 93.21%, F1-Score: 93.27%	English
Yao et al. (2020)	Reddit users from subreddits related to suicide, depression, opioids, and control subreddits	(C1): (Training: 41,092 posts—80%; Testing: 10,274 posts—20%)	Machine Learning–Based Approach	Accuracy: 95.4%, Precision: 96.8%, Recall: 95.3%, F1-Score: 96.1%	English
Boonyarat et al. (2024)	Thai X users	2,400 annotated tweets evaluated with 10-fold cross-validation.	Linguistic Feature-infused BERT (LFBERT)	Precision: 92%, Recall: 93%, F1-Score: 93%	Thailand
Chatterjee et al. (2023)	2,000 Twitter users (posts about depression)	Total: 188,704 posts	Mental Health Curve	Accuracy: 89%, Precision: 88%, Recall: 87%, F1-Score: 88%	English
		Training: 150,964 posts—80%			
		Testing: 37,740 posts—20%			
Xie-Yi (2024)	Tweets expressing suicidal or non-suicidal ideation	X users: not specified	Bi-LSTM with attention layer (SID: Suicidal Ideation Detection)	Accuracy: 94%, Precision: 95%, Recall: 93%, F1-Score: 94%	English
Mirtaheri et al. (2024)	X and Reddit users	X_1 = 9,119, X_2 = 17,142, Reddit_SNS = 232,074, Total = 258,335	*AL-BTCN*	Accuracy: 95%, Precision: 95%, Recall: 94%, F1-Score: 95%	English
Anika et al. (2024)	Reddit and X users	Reddit: 232,074 posts (116,037 suicidals, 116,037 non-suicidals), X: 9,119 tweets (3,998 suicidals, 5,121 non-suicidals)	Hybrid BiGRU-CNN	Accuracy: 93.07%, Precision: 93%, Recall: 94%, F1-Score: 93%	English
Malhotra and Jindal (2024)	Users of Social media X and Reddit	X: 23,520, Reddit: 231,967, X: 5,540, Reddit: 1,895, 80% training, 10% validation, and 10% testing.”	Integrated Gradients + NLG (XAI-Transformer)	Precision: 97.0%, Recall: 96.4%, F1-Score: 96.7%, Accuracy: 96.7%	English
Gorai and Shaw (2024)	Social media users (X and Reddit) and real-life suicide notes	X+ CEASE: 12,061, Reddit+ CEASE: 6,390	BERT-encoded ensembled CNN model	Precision: 98.1%, Recall: 97.9%, F1-Score: 98.0%, Accuracy: 99.5%	English
Ezerceli and Dehkharghani (2024)	Reddit users and suicide notes	SuicideDetection: 20,000 posts CEASE-v2.0 training (70%), validation (10%), and testing (20%) sets	LSTM y CNN	Precision: 97.0%, Recall: 97.0%, F1-Score: 97.0%, Accuracy: 98.0%	English
Kancharapu and Ayyagari (2024)	X users	148,768 tweets, After filtering, 20,000 posts	GAN-Infused Deep Learning Framework with Genetic Optimization and Word Embedding Fusion	Precision: 97%, Recall: 97%, F1-Score: 97%	English
		Training 80%, testing 20%			
Qorich and El Ouazzani (2024)	Reddit users	Reddit:232,074 posts, Training 75%, testing 25%	C-BiLSTM model using triple word embedding	Accuracy: 94.95%, F1-score: 94.95%	English
Kodati and Tene (2024)	Reddit users and suicide note	Reddit: 6,820, CEASE-v2.0: 4,932	CoDyn-BMHSA-CNN	Accuracy: 97.4%, F1-score: 93.8%, Precision: 94.1%, Recall: 92.7%	English
		Training 80%, testing 20%			
Bokhari et al. (2024)	Indian population data (state, year, suicide type, gender, age)	Suicide_dataset.csv: 235,000, Training 80%, testing 20%	Neural Network Ensemble	Accuracy: 91.0%, Precision: 90.0%, Recall: 86.0%, F1-score: 88.0%, ROC AUC: 93.0%	English
Pillai et al. (2024)	Voice recordings from individuals with MDD, AVH, PT, and students with suicidal thoughts.	106 depression (AVEC2019), 64 suicidal students (SafeAudio), 102 suicide risk (SuicideRisk), 46 AVH/PT (U-Safe) total: 318 participants	S3R (proposed) voice detection method	Accuracy: 35%, F1-Score: 34%	English
Tlachac et al. (2022)	Undergraduate and graduate students	302 students	StudentSADD	Accuracy: 78%, F1-Score: 70.6%, Precision: 66%, Recall: 76%	English
Ahmed et al. (2023)	Reddit users	15,044 Reddit posts, 14,944 used for training	Graph Attention Network (GAT)	Precisio: 89%, Recall: 90%, F1-Score: 89%	English
				ROC-AUC: 91%	
Kumar et al. (2023)	Reddit users and social media posts in Arabic and English,	Dataset D1 (Arabic): 976 training + 245 testing posts, Dataset D2 (Arabic): 846 training + 212 testing posts, Dataset D3 (English): 8891 training + 4,496 testing posts,	AC-BERT + Bi-LSTM fine-tuned RoBERTa	*AC-BERT + Bi-LSTM Arabic language:* Accuracy: 82%, F1-score: 82%, Precision: 82%, Recall: 82%	Arabic, English
				Fine tuned	
				*RoBERTa English language:* Accuracy: 61%, F1-score: 60%, Precision: 61%, Recall: 61%	
Xu et al. (2024)	Reddit users from multiple mental health subreddits	Dreaddit, 3,553, DepSeverity, 3,553, SDCNL, 1895, CSSRS-Suicide (Tarea 5), 500, CSSRS-Suicide (Tarea 6), 500, 80% training, 20% test	Large Language Models (LLMs) instruction finetuning	Balanced accuracy: 86.8%	English

### 3.5 Detected trends

There are significant trends in the computational methods used to detect suicidal ideation in texts. For example, models that use transformer architectures, such as BERT and GPT, are distinguished by their ability to understand linguistic context deeply, achieving accuracy of up to 97.6% in social media posts. It is important to mention that BERT and GPT are models based on transformer architectures that use attention mechanisms as a central component to model contextual relationships in text.

The subgroup study showed that recurrent neural networks (RNNs) are particularly efficient at processing short and emotional texts, and that models based on transformer architectures that incorporate attention mechanisms excel at complex semantic analysis. The combination of precision and F1-score metrics shows that hybrid approaches that integrate techniques such as CNN and LSTM with linguistic rules offer robust and adaptable solutions, especially in contexts with limited data or high noise levels, due to the ability to overcome the limitations of individual models by integrating complementary approaches, thus taking advantage of the specific strengths of each.

Beyond the classic approaches, this review also identified studies with great potential. Some studies applied generative adversarial networks (GANs) to generate synthetic examples of data with suicidal ideation, improving the diversity of the data, especially when the corpus was limited. Large-scale language models such as ChatGPT or Flan-T5 were also used to aid data generation and classification tasks.

Other studies explored approaches such as graph neural networks, which allow for better representation of relationships between words within a text. Some studies combined voice and text, achieving multimodal models with good results in more complex contexts. Although they do not have metrics close to 100%, they are a major advance given the type of data handled, such as voice notes in which the aim was to identify signs of suicidal ideation. Finally, ensemble methods combine different models such as SVM, CNN, or Random Forest, which can combine various technologies to overcome the individual shortcomings that each method may have if worked individually.

### 3.6 Reporting biases

To assess potential reporting biases, we identified a predominance of indexed academic databases, which may limit the information’s cultural and contextual diversity by excluding gray literature. We also found a clear linguistic bias toward English-language datasets, restricting the applicability and generalization of the findings to multilingual contexts. We also observed a heavy reliance on data primarily from Reddit, the social network X, and other similar platforms that exhibit specific communication and interaction patterns. However, this choice of sources is partly due to acceptance criteria, higher methodological quality, and the availability of labelled data. Furthermore, studies that used sources other than social media, such as clinical notes or voice recordings, were included, and some studies combined multiple datasets. The strategies described partially mitigated the impact of biases and strengthened the validity of the findings.

## 4 Discussion

### 4.1 Synthesis of results

The systematic review revealed various computationally efficient approaches for detecting suicidal thoughts in natural language texts. They include deep learning techniques, large-scale language models (LLMs), ensemble methods, and advanced approaches such as GNNs, GANs, and multimodal models. Beyond the diversity of computational approaches identified, a cross-study analysis indicates that no single model consistently outperforms others across all contexts. Instead, model performance is strongly conditioned by factors such as dataset size, linguistic characteristics, annotation quality, and the explicitness of suicidal expressions. Studies using larger and more heterogeneous datasets tend to favor transformer-based architectures, while those relying on smaller or more controlled corpora often report competitive results with traditional deep learning or hybrid models. This pattern suggests that reported performance should be interpreted in relation to methodological context rather than model choice alone.

Deep learning presents architectures such as LSTM, Bi-LSTM, and CNN with the ability to perform long-term sequences and dependencies, as well as to capture complex semantic patterns (Schoene et al., 2023b; Boonyarat et al., 2024; Bokhari et al., 2024). By incorporating attention mechanisms, the models’ accuracy and interpretability are improved (Xie-Yi, 2024; Gorai and Shaw, 2024). Similarly, it was shown that convolutional networks show good performance on platforms such as Reddit, and hybrid CNN-BiGRU models allow refining the results by combining local and contextual features (Ezerceli and Dehkharghani, 2024; Mirtaheri et al., 2024). A comparative analysis of these studies suggests that deep learning architectures such as LSTM and CNN are particularly effective when suicidal ideation is expressed through explicit lexical or sequential patterns. However, their performance decreases when dealing with highly implicit, metaphorical, or context-dependent language, especially in short texts. Furthermore, studies that incorporate attention mechanisms or hybrid configurations tend to report more stable results, indicating that architectural enhancements play a crucial role in mitigating the limitations of standard deep learning models.

Large-scale language models, such as BERT, RoBERTa, and GPT, specialize in detecting explicit and implicit cues associated with suicidal ideation. Their ability to illustrate context in detail facilitates more accurate identification of risky expressions, even in ambiguous texts. Mixtures of Transformers and Bi-LSTMs, particularly in multilingual environments, are beneficial in identifying suicidal ideation (Qorich and El Ouazzani, 2024). Despite their superior contextual modeling capabilities, large-scale language models present important practical and methodological challenges. Their effectiveness is closely linked to the availability of large, annotated datasets and significant computational resources, which may limit their applicability in low-resource or real-time clinical settings. Additionally, several studies highlight concerns regarding model interpretability, as transformer-based architectures often function as black boxes, complicating their integration into mental health decision-making processes where transparency and accountability are essential.

Regarding the ensemble procedures, it was found that structures such as Random Forest, SVM, k-NN, and CNN have achieved metrics exceeding 90% in classification tasks (Yao et al., 2020; Vioulès et al., 2018). Combining models such as BERT with CNN, or using convolutional temporal networks with self-attention, has facilitated handling significant and challenging data sets with superior results (Gorai and Shaw, 2024; Schoene et al., 2023a). However, traditional algorithms have restrictions when compared with more current models, particularly in contexts of emotional or ambiguous language (Xie-Yi, 2024; Tlachac et al., 2022). Although ensemble-based approaches frequently report high performance metrics, including accuracy values exceeding 90%, these results should be interpreted with caution. A closer examination reveals that many of these studies rely on limited datasets, single-platform sources, or lack rigorous validation strategies such as cross-validation or external testing. Consequently, high numerical performance does not necessarily reflect model robustness or generalizability, underscoring the importance of methodological rigor over metric optimization alone.

Finally, advanced approaches show great potential, for example, GNNs have been used to model complex semantic relationships, while GATs have improved the categorization of suicidal ideas in social media data (Xie-Yi, 2024; Priyamvada et al., 2023). GANs have facilitated the creation of artificial data to cover the lack of labelled data, thus improving the performance of models in contexts with restricted information (Schoene et al., 2023b; Kancharapu and Ayyagari, 2024). Multimodal models that fuse text and speech have also demonstrated remarkable effectiveness, particularly when incorporating networks such as VGG and BERT for the study of voice and language, considering that it is a much greater challenge to identify suicidal ideas in this type of data given its complexity (Anika et al., 2024; Chatterjee et al., 2023; Belouali et al., 2021). From a cross-study perspective, advanced approaches such as GNNs, GANs, and multimodal architectures demonstrate significant potential but also introduce increased complexity. While these models enhance semantic representation and data diversity, they require substantial computational resources and sophisticated data preprocessing pipelines. As a result, their implementation may be more suitable for research or specialized clinical environments rather than large-scale or resource-constrained applications, highlighting a trade-off between performance gains and practical feasibility.

This systematic review contributes new insights to the field by identifying methodological gaps in current approaches and highlighting how recent advances in artificial intelligence, such as Large Language Models (LLMs), Generative Adversarial Networks (GANs), and Graph Neural Networks (GNNs), are being applied to suicidal ideation detection. Unlike previous reviews that primarily focus on describing individual techniques or specific model families, this synthesis integrates a critical analysis of these advances and offers an updated perspective on computational approaches, including practical implications for their implementation in real-world suicide prevention contexts.

### 4.2 Limitations

This systematic review presents limitations at two levels: those inherent to the included studies and those related to the review process itself. Regarding the limitations of the reviewed studies, a recurring constraint is the lack of linguistic diversity in the datasets used. Most models were trained with data in English, which restricts their applicability to different cultural and linguistic contexts (Malhotra and Jindal, 2024; Xu et al., 2024; Qorich and El Ouazzani, 2024). Furthermore, many studies depend on sources such as Reddit or social network X, which generate population and expression biases by not having more diverse data sources to conduct their tests.

These limitations have direct implications for the validity and transferability of the reported findings. Models trained predominantly on English-language and platform-specific data may inadvertently learn linguistic or behavioral patterns that are not representative of broader populations. This can lead to overfitting, reduced sensitivity to culturally implicit expressions of distress, and inflated performance estimates when applied to new contexts.

Other studies show methodological flaws, such as the use of small samples, lack of cross-validation, or limited clarity in the structure of the models. These restrictions complicate the contrast between approaches and reduce the extrapolation of findings (Yao et al., 2020; Kodati and Tene, 2024; Vioulès et al., 2018). Additionally, the heterogeneity of the included studies regarding data sources, computational model architectures, sample sizes (ranging from 302 to 258,335 records), and the diversity of evaluation metrics employed did not allow for a quantitative meta-analysis of the resulting data.

Concerning the limitations of the review process, significant challenges were encountered in developing search strategies. Multiple adjustments were necessary to include relevant studies that did not use the most common keywords. Furthermore, the number of available studies on the automatic detection of suicidal ideation using NLP remains low compared to other areas in the field. Difficulties in data extraction were also identified because the studies did not present their results similarly.

### 4.3 Implications

The findings of this systematic review have significant implications for multiple stakeholders involved in suicide prevention, including technology developers, mental health professionals, and public health policymakers. From a technological perspective, the demonstrated effectiveness of transformer-based architectures, particularly BERT and its variants, achieving accuracy rates up to 97.6% in social media analysis, suggests that these models are sufficiently mature for integration into early warning systems. The superior performance of hybrid approaches combining CNN and LSTM with attention mechanisms indicates that future tool development should prioritize architectural flexibility over single-model solutions. Furthermore, the success of ensemble methods, which consistently achieved metrics exceeding 90%, demonstrates that combining multiple algorithms can effectively compensate for individual model limitations, particularly in contexts characterized by ambiguous or emotionally complex language. For mental health professionals, the computational approaches identified in this review offer promising opportunities to extend screening capabilities beyond clinical settings. The predominant use of Reddit (65.2%) and social network X (33.2%) as data sources reflects the potential of these platforms as environments where individuals may express distress signals that would otherwise remain undetected. However, the low representation of clinical data (1.4%) indicates a critical gap between research developments and clinical applicability. Mental health practitioners should be aware that current models are primarily trained on social media language patterns, which may differ substantially from clinical interview contexts or written patient communications. The geographical concentration of research, with India contributing 30% of the studies followed by the United States and United Kingdom at 15% each, has important implications for global applicability. This distribution suggests that computational suicide prevention tools are being developed predominantly within specific cultural and linguistic frameworks. The near-exclusive reliance on English-language datasets means that the linguistic markers and expression patterns learned by these models may not generalize to populations communicating in other languages or cultural contexts. For public health systems in non-English-speaking regions, this represents both a limitation and an opportunity for localized model development. Regarding model interpretability, the review revealed that while transformer-based models achieve high performance metrics, their decision-making processes often remain opaque. This presents a significant barrier for clinical adoption, where understanding why a model flags particular content as indicative of suicidal ideation is essential for appropriate intervention. The emerging application of explainable AI (XAI) approaches, as demonstrated by Malhotra and Jindal (2024), represents a critical direction for making these tools acceptable within mental health practice, where accountability and transparency are paramount. The ethical implications of deploying computational methods for suicidal ideation detection warrant careful consideration. The analysis of social media posts without direct user interaction raises questions about privacy, consent, and the potential consequences of false positives and false negatives. A false positive may result in unnecessary interventions or stigmatization, while a false negative could mean missing individuals in genuine crisis. The high recall rates reported by several models (exceeding 93% in some cases) suggest prioritization of sensitivity over specificity, which may be appropriate given the severe consequences of missed detections in this domain.

### 4.4 Future work

Based on the synthesized evidence, future research should prioritize methodological robustness alongside model innovation. The development of multilingual and culturally diverse datasets represents a fundamental need, as current models trained predominantly on English-language data cannot adequately serve global populations (Malhotra and Jindal, 2024; Xu et al., 2024; Qorich and El Ouazzani, 2024).

The integration of multiple data sources, including text, voice, and images, should be expanded to capture the multimodal nature of human communication. Multimodal models have already demonstrated significant progress in this direction (Anika et al., 2024; Chatterjee et al., 2023; Belouali et al., 2021), and further development could enable more comprehensive risk assessment.

Improving model interpretability remains essential for clinical adoption. Progressing toward more transparent, understandable, and ethically viable AI systems will require interdisciplinary cooperation between mental health experts, linguists, and technology developers. Such collaboration is necessary to develop solutions that are both technically effective and practically implementable in real-world healthcare settings (Ahmed et al., 2023; Ghanadian et al., 2024; Tlachac et al., 2022).

Additionally, the use of synthetic data generated with Large Language Models offers a promising approach to address data scarcity while maintaining ethical standards, and the development of more explainable models (XAI) should continue to bridge the gap between computational performance and clinical utility.

## 5 Conclusion

This paper presents a systematic review of the literature using the PRISMA 2020 method (Page et al., 2021), analyzing 25 studies on detecting suicidal ideation through computational methods. The results show essential advances, especially in using hybrid models and Large Language Models (LLMs) such as BERT, RoBERTa, and GPT. Together with architectures such as CNN, LSTM, and BiGRU, these models have achieved strong results in metrics such as Accuracy, Precision, Recall, and F1-score, demonstrating their potential to detect suicidal ideation even outside clinical contexts.

An essential aspect for future studies is the ethical dimension of using computational models to detect suicidal ideation. The collection and analysis of personal data raise sensitive issues regarding privacy, consent, and responsible use of results, especially when working with social media posts without direct interaction with users. Ensuring the integrity and protection of assessed individuals must remain a priority in this field.

Despite these advances, important challenges remain. As discussed in Section 4.4, key areas requiring attention include expanding linguistic and cultural diversity in datasets, improving model interpretability for clinical adoption, and fostering interdisciplinary collaboration. Promising proposals for addressing these limitations include the use of synthetic data generated with LLMs, cross-validation techniques, and the development of more explainable models (XAI).

In summary, computational tools demonstrate strong performance in automating the identification of suicidal ideation in natural language texts, indicating their potential as effective instruments for early suicide prevention. Overcoming the identified limitations will be critical for translating these approaches into practical applications that contribute meaningfully to suicide prevention strategies worldwide.

## Data Availability

The original contributions presented in the study are included in the article/supplementary material, further inquiries can be directed to the corresponding author.
